# The Twenty-Second Annual Convention of the Texas State Medical Association

**Published:** 1890-04

**Authors:** 


### EDITORIAL DEPARTMENT.

F. E. DANIEL, M. D., EDITOR AND PUBLISHER.

THIS JOURNAL, by unanimous vote of the members, was made the Official Organ of the Austin District Medical Society.

COLLABORATORS:

*Dr. R. M. Swearingen, Austin.*
*Prof. B. E. Hadra, M. D., Galveston.*
*Prof. Geo. Cupples, M. D., San Antonio.*
*Dr. T. C. Osborn, Cleburne, Texas.*
*Dr E. J. Doering, Chicago.*
*Dr. E. J. Beall, Fort Worth.*
*Dr Odo Betz, Germany.*
*Dr. J. W. McLaughlin, Austin.*
*Dr. T. J. Tyner, Austin.*
*Prof. J. F. Y. Paine, M. D., Galveston.*
*Dr. R. H. L. Bibb, Mexico.*
*Dr. T. J. Bennett. Austin.*
*Dr. Bat Smith, Wharton.*
*Dr. E. Meierhof, New York.*
*Prof. W. B. Rogers, M. D., Memphis, Tenn.*

### THE TWENTY-SECOND ANNUAL CONVENTION OF THE TEXAS STATE MEDICAL ASSOCIATION.

At the meeting to be held at Fort Worth the last week in April (inst.), beginning Tuesday, 22nd, and holding four days, the Texas State Medical Association will attain its majority—will be just twenty-one years of age. It was organized in Houston, in April, 1869; by a few devoted followers of Æsculapius. Alas, how few are left of them to celebrate the Association's twenty-first birth-day ! We can recall of the charter members only Drs. D. R. Wallace, T. J. Heard, D. F. Stuart and R. T. Flewellyn!—there may be others but we do not recall them—who officiated at the birth of this now strong young man-society,—so full of vim and vigor, and good, we hope, for fifty years more of useful existence. We suggest to the committee of arrangement that something more than ordinary ought to be done, to commemmorate this important event. The Association's attainment of legal age should be recognized in an appropriate manner; it would have been well to defer the chartering of the body till now; but that is past.

For the information of those who contemplate applying for membership and who have not heretofore attended the meetings, we publish below the requirements for membership, and the objects for which the Association was formed; together with a list of the officers of Sections, for the benefit of those who desire to contribute papers.

Article 3 of the Constitution says: "Every regularly educated man within the limits of the State, who is a graduate of a regular medical college in good standing, and who adopts and conforms to the code of ethics of the American Medical Association, shall be eligible to membership in this body; provided they are members in good standing in their respective county or district medical societies.

II. "In all cases where there exists no county medical association (and the qualifications for membership are perfect in every other respect) this fact shall not be a bar to membership.

[Applicants for membership must show a diploma, or certificate that diploma has been registered, and be endorsed by two members.—ED.]

Article 2 of the Constitution says: "The objects of this Association shall be to organize the medical profession of the State in the most efficient manner possible; to encourage a high standard of qualification and ethics; to promote professional brotherhood; to labor for advancement of State medicine, i. e., of public hygiene; of medical education; of medical jurisprudence and public institutions for the sick and the infirm.

The Judicial Council consists of Drs. P. C. Coleman, R. Rutherford, M. D. Knox, R. C. Nettles, I. E. Clarke, C. M. Ramsdell, T. J. Bell, R. H. Harrison, Sr., F. S. White, E. M. Rabb, J. V. Spring and T. J. Wagley.

The Officers are: President, R. M. Swearingen; Vice Presidents, A. Sims, B. F. Kingley and I. E. Clarke; Secretary, F. E. Daniel; Treasurer, J. Larendon.

Section on Practice—Dr. D. M. Ray, Chairman; Dr. O. Eastland, Secretary.

Section on Obstetrics—Dr. S. D. Thruston, Chairman.

Section on Surgery and Anatomy—Dr. W. L. York, Chairman.

Section on State Medicine, etc.—Dr. E. L. Ward, Chairman.

Section on Gynecology—Dr. H. K. Leake, Chairman.

Section on Medical Jurisprudence—Dr. A. D. Burroughs, Chairman.

Section on Ophthalmalogy and Otology—Dr. G. P. Hall, Chairman.

Section on Electro-Therapeutics—Dr. A. F. Sampson, Chairman.

Section on Dermatology—Dr. S. H. Weatherford, Chairman.

Committee of Arrangements—Dr. W. P. Burts, Chairman.

Committee on Necrology—Dr. B. F. Britain, Chairman.

Let all members realize that an imperative duty requires that they should make an effort to attend this meeting. We are engaged in a grand work—one of regeneration and progress, and need the co-operation of every member. Let them also use their influence to induce others—not members—to come and join,—to bring into the fold all the desirable men in the State to help in the great work. We have an Augean stable to clean out, and neither Hercules nor the Euphrates to help us. We must apply ourselves to the task and determine to succeed,—we must reclaim the State; it is overrun with quacks.

At this meeting, among other things, it will be necessary to appoint delegates to the convention in Washington, May 7, to revise the U. S. Pharmacopœia. If any member would like to go, let him make it known to the nominating committee.

Since last meeting the Association has lost some of its oldest members, to wit:

Dr. J. B. Robertson, of Goliad;

Dr. Ed. Randall, of Galveston;

Drs. A. E. and E. J. Carothers, of San Antonio;

Dr. J. M. Lewis, of Mexia, and

Dr. B. B. White, of Ennis.

Peace to their ashes.

The President's address will be a *gem*, and alone worth the expense and time incident to leaving home to attend the meeting. Let all lay down their work for four days and swell the ranks of the big convention,—and last, but not least—let them bring a little extra cash, to renew their subscription to the JOUR-

NAL, and to pay up their dues; the latter is *imperative*, as payment must be made on registry.

